# Methyl 2,2′-dimethyl-4′-[2-(methyl­sulfan­yl)eth­yl]-1,3-dioxo-2,3-dihydro-1*H*,4′*H*-spiro­[isoquinoline-4,5′-oxazole]-4′-carboxyl­ate

**DOI:** 10.1107/S1600536811030133

**Published:** 2011-08-02

**Authors:** Hoong-Kun Fun, Ching Kheng Quah, Chengmei Huang, Haitao Yu

**Affiliations:** a X-ray Crystallography Unit, School of Physics, Universiti Sains Malaysia, 11800 USM, Penang, Malaysia; b School of Chemistry and Chemical Engineering, Nanjing University, Nanjing 210093, People’s Republic of China

## Abstract

In the isoquinoline ring system of the title mol­ecule, C_18_H_20_N_2_O_5_S, the fused *N*-heterocyclic ring is distorted towards a half-boat conformation. The methyl formate moiety is disordered over two sets of sites with refined occupancies of 0.882 (5) and 0.118 (5). In the crystal, mol­ecules are linked *via* weak inter­molecular C—H⋯O hydrogen bonds into one-dimensional chains along [010].

## Related literature

For general background to and the biological activity of isoquinoline- and oxazole-containing compounds, see: Yu *et al.* (2010 ▶); Huang *et al.* (2011 ▶); Harris *et al.* (2005 ▶); Vintonyak *et al.* (2010 ▶); Badillo *et al.* (2010 ▶, 2011 ▶); Wang *et al.* (2010 ▶); Nair *et al.* (2002 ▶). For the stability of the temperature controller used for the data collection, see: Cosier & Glazer (1986 ▶). For standard bond-length data, see: Allen *et al.* (1987 ▶). For ring conformations, see: Cremer & Pople (1975 ▶). For related structures, see: Fun *et al.* (2011*a* ▶,*b* ▶,*c* ▶,*d* ▶).
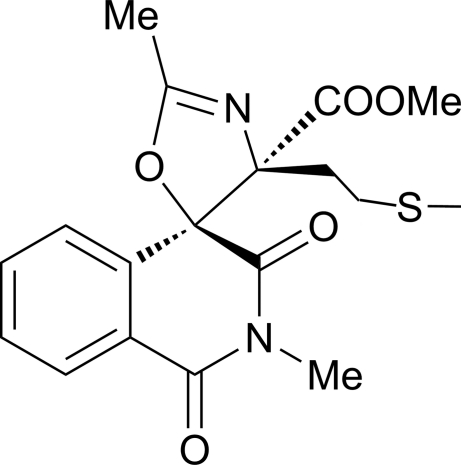


## Experimental

### 

#### Crystal data

C_18_H_20_N_2_O_5_S
*M* *_r_* = 376.42
Monoclinic, 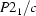

*a* = 15.0052 (15) Å
*b* = 8.4548 (8) Å
*c* = 15.4915 (15) Å
β = 114.621 (2)°
*V* = 1786.7 (3) Å^3^
*Z* = 4
Mo *K*α radiation
μ = 0.21 mm^−1^
*T* = 100 K
0.18 × 0.17 × 0.14 mm

#### Data collection

Bruker APEXII DUO CCD area-detector diffractometer
Absorption correction: multi-scan (*SADABS*; Bruker, 2009 ▶) *T*_min_ = 0.963, *T*_max_ = 0.971
14571 measured reflections
4063 independent reflections
3343 reflections with *I* > 2σ(*I*)
*R*_int_ = 0.046

#### Refinement

*R*[*F* ^2^ > 2σ(*F* ^2^)] = 0.042
*wR*(*F* ^2^) = 0.120
*S* = 1.03
4063 reflections
251 parameters
5 restraints
H-atom parameters constrained
Δρ_max_ = 0.37 e Å^−3^
Δρ_min_ = −0.32 e Å^−3^

### 

Data collection: *APEX2* (Bruker, 2009 ▶); cell refinement: *SAINT* (Bruker, 2009 ▶); data reduction: *SAINT*; program(s) used to solve structure: *SHELXTL* (Sheldrick, 2008 ▶); program(s) used to refine structure: *SHELXTL*; molecular graphics: *SHELXTL*; software used to prepare material for publication: *SHELXTL* and *PLATON* (Spek, 2009 ▶).

## Supplementary Material

Crystal structure: contains datablock(s) global, I. DOI: 10.1107/S1600536811030133/lh5290sup1.cif

Structure factors: contains datablock(s) I. DOI: 10.1107/S1600536811030133/lh5290Isup2.hkl

Supplementary material file. DOI: 10.1107/S1600536811030133/lh5290Isup3.cml

Additional supplementary materials:  crystallographic information; 3D view; checkCIF report

## Figures and Tables

**Table 1:** Hydrogen-bond geometry (Å, °)

*D*—H⋯*A*	*D*—H	H⋯*A*	*D*⋯*A*	*D*—H⋯*A*
C18—H18*C*⋯O2^i^	0.96	2.49	3.436 (2)	167

Symmetry code: (i) 
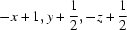
.

**Fig. 1.**
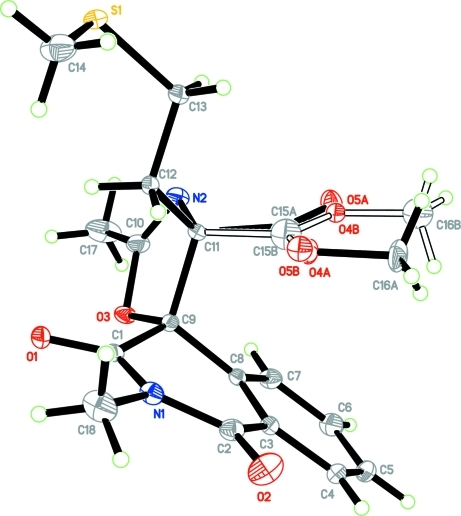
The molecular structure of the title compound showing 30% probability displacement ellipsoids for non-H atoms. The minor component of disorder is shown as open bonds.

**Fig. 2.**
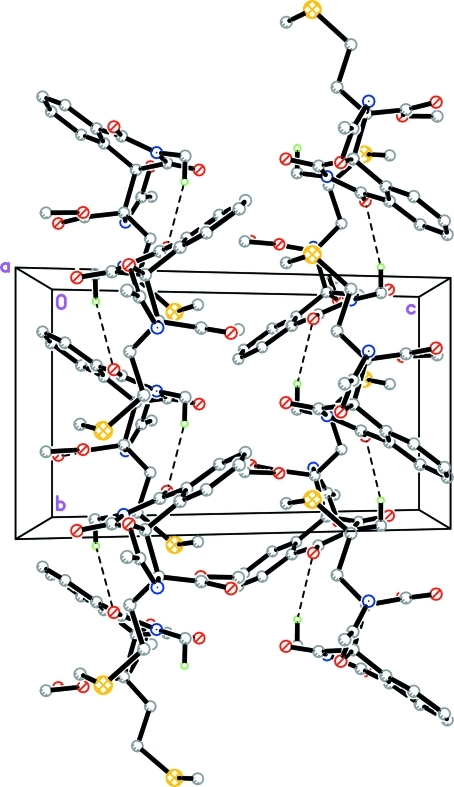
Part of the crystal structure of the title compound, viewed along the a axis. H atoms not involved in hydrogen bonds (dashed lines) have been omitted for clarity. Only the major disorder component is shown.

## supplementary crystallographic information

### Comment

Photocycloaddition of isoquinoline-1,3,4-trione combined with following transformation of the photocycloadducts has become facile method to build various scaffold containing isoquinoline moiety (Yu *et al.*, 2010; Huang *et al.*, 2011). Oxazoles can be used to inhibit the activity of malignant tumors (Harris *et al.*, 2005). Spirocyclic oxindoles have emerged as attractive synthetic targets because of their prevalence in numerous natural products and important biological activity (Badillo *et al.*, 2010; Vintonyak *et al.*, 2010). Among them, the synthesis of spirooxindole oxazoles is of great intrest (Badillo *et al.*, 2011; Wang *et al.*, 2010; Nair *et al.*, 2002). Many bioactive natural products especially alkaloids contain an isoquinoline or oxazole ring. It is necessary to develop methodologies to construct such moieties. The title compound which was derived from isoquinoline-1,3,4-trione and an oxazole and may have potential use in biochemical and pharmaceutical fields.

In the racemic title compound, Fig. 1, atoms C9 and C11 are the chiral centers. The isoquinoline ring system (N1/C1-C9) is not completely planar, the *N*-heterocyclic ring (N1/C1-C3/C8/C9) being distorted towards a half-boat conformation with atom C9 deviating by 0.213 (2) Å from the mean plane through the remaining atoms, puckering parameters (Cremer & Pople, 1975) Q = 0.3237 (18) Å, Θ =  67.0 (3)° and φ = 102.1 (3)°. Bond lengths (Allen *et al.*, 1987) and angles are within normal ranges and comparable to related structures (Fun *et al.*, 2011*a*, *b*, *c*, *d*). The methyl formate moiety (O4/O5/C15/C16) is disordered over two positions with refined site-occupancies of 0.882 (5) and 0.118 (5).

In the crystal, Fig. 2, molecules are linked *via* intermolecular C18–H18C···O2^i^ hydrogen bonds (Table 1) into infinite one-dimensional chains along [010].

### Experimental

The title compound was the main product from the acid-catalyzed transformation of the photocyclo adduct of isoquinoline-1,3,4-trione and 4-(2-(methylthio)ethyl)-5-methoxy-2-methyloxazole. The compound was purified by flash column chromatography with ethyl acetate/petroleum ether (1:4) as eluents. X-ray quality crystals of the title compound were obtained from slow evaporation of an acetone and petroleum ether solution (1:5) of the title compound (*m.p.* 440-142 K).

### Refinement

All H atoms were positioned geometrically and refined using a riding model with C–H = 0.93 - 0.97 Å and *U*_iso_(H) = 1.2 or 1.5 *U*_eq_(C). The highest residual electron density peak is located at 0.76 Å from C2 and the deepest hole is located at 0.70 Å from S1. The same U^ij^ parameters were used for atom pair C15B/C16B. The methyl formate moiety (O4/O5/C15/C16) is disordered over two positions with refined site-occupancies of 0.882 (5) : 0.118 (5).  All minor disordered components were refined isotropically.

### Crystal data

**Table:**

C_18_H_20_N_2_O_5_S	*F*(000) = 792
*M**_r_* = 376.42	*D*_x_ = 1.399 Mg m^−^^3^
Monoclinic, *P*2_1_/*c*	Mo *K*α radiation, λ = 0.71073 Å
Hall symbol: -P 2ybc	Cell parameters from 4051 reflections
*a* = 15.0052 (15) Å	θ = 2.8–32.2°
*b* = 8.4548 (8) Å	µ = 0.21 mm^−^^1^
*c* = 15.4915 (15) Å	*T* = 100 K
β = 114.621 (2)°	Block, colourless
*V* = 1786.7 (3) Å^3^	0.18 × 0.17 × 0.14 mm
*Z* = 4	

### Data collection

**Table:**

Bruker APEXII DUO CCD area-detector diffractometer	4063 independent reflections
Radiation source: fine-focus sealed tube	3343 reflections with *I* > 2σ(*I*)
graphite	*R*_int_ = 0.046
φ and ω scans	θ_max_ = 27.5°, θ_min_ = 2.8°
Absorption correction: multi-scan (*SADABS*; Bruker, 2009)	*h* = −19→19
*T*_min_ = 0.963, *T*_max_ = 0.971	*k* = −10→10
14571 measured reflections	*l* = −20→11

### Refinement

**Table:**

Refinement on *F*^2^	Primary atom site location: structure-invariant direct methods
Least-squares matrix: full	Secondary atom site location: difference Fourier map
*R*[*F*^2^ > 2σ(*F*^2^)] = 0.042	Hydrogen site location: inferred from neighbouring sites
*wR*(*F*^2^) = 0.120	H-atom parameters constrained
*S* = 1.03	*w* = 1/[σ^2^(*F*_o_^2^) + (0.0626*P*)^2^ + 0.5509*P*] where *P* = (*F*_o_^2^ + 2*F*_c_^2^)/3
4063 reflections	(Δ/σ)_max_ = 0.001
251 parameters	Δρ_max_ = 0.37 e Å^−^^3^
5 restraints	Δρ_min_ = −0.32 e Å^−^^3^

### Special details

**Table:**

Experimental. The crystal was placed in the cold stream of an Oxford Cryosystems Cobra open-flow nitrogen cryostat (Cosier & Glazer, 1986) operating at 100.0 (1) K.
Geometry. All esds (except the esd in the dihedral angle between two l.s. planes) are estimated using the full covariance matrix. The cell esds are taken into account individually in the estimation of esds in distances, angles and torsion angles; correlations between esds in cell parameters are only used when they are defined by crystal symmetry. An approximate (isotropic) treatment of cell esds is used for estimating esds involving l.s. planes.
Refinement. Refinement of F^2^ against ALL reflections. The weighted R-factor wR and goodness of fit S are based on F^2^, conventional R-factors R are based on F, with F set to zero for negative F^2^. The threshold expression of F^2^ > 2sigma(F^2^) is used only for calculating R-factors(gt) etc. and is not relevant to the choice of reflections for refinement. R-factors based on F^2^ are statistically about twice as large as those based on F, and R- factors based on ALL data will be even larger.

### Fractional atomic coordinates and isotropic or equivalent isotropic displacement parameters (Å^2^)

**Table:**

	*x*	*y*	*z*	*U*_iso_*/*U*_eq_	Occ. (<1)
S1	0.27696 (3)	1.10762 (5)	0.32691 (3)	0.02485 (13)	
O1	0.33441 (10)	0.50297 (14)	0.39092 (8)	0.0279 (3)	
O2	0.46168 (9)	0.36107 (18)	0.18390 (11)	0.0383 (3)	
O3	0.14782 (8)	0.46617 (13)	0.26111 (8)	0.0221 (3)	
N1	0.39944 (10)	0.44834 (16)	0.28567 (10)	0.0217 (3)	
N2	0.10538 (10)	0.70860 (16)	0.19482 (10)	0.0235 (3)	
C1	0.32154 (12)	0.47773 (18)	0.30951 (11)	0.0194 (3)	
C2	0.38950 (12)	0.38490 (19)	0.19925 (12)	0.0231 (3)	
C3	0.28965 (11)	0.33921 (18)	0.13157 (11)	0.0185 (3)	
C4	0.27876 (13)	0.24490 (19)	0.05343 (12)	0.0244 (4)	
H4A	0.3335	0.2156	0.0435	0.029*	
C5	0.18662 (14)	0.1955 (2)	−0.00886 (12)	0.0295 (4)	
H5A	0.1792	0.1324	−0.0606	0.035*	
C6	0.10523 (14)	0.2401 (2)	0.00594 (13)	0.0306 (4)	
H6A	0.0433	0.2056	−0.0357	0.037*	
C7	0.11511 (12)	0.3360 (2)	0.08231 (12)	0.0248 (4)	
H7A	0.0599	0.3668	0.0910	0.030*	
C8	0.20750 (11)	0.38568 (17)	0.14568 (11)	0.0168 (3)	
C9	0.22153 (11)	0.49560 (18)	0.22694 (11)	0.0168 (3)	
C10	0.08495 (13)	0.5926 (2)	0.23464 (13)	0.0257 (4)	
C11	0.20146 (11)	0.67886 (18)	0.19522 (11)	0.0176 (3)	
C12	0.27506 (12)	0.79601 (18)	0.26518 (11)	0.0201 (3)	
H12A	0.2747	0.7840	0.3273	0.024*	
H12B	0.3405	0.7719	0.2710	0.024*	
C13	0.24935 (14)	0.96755 (19)	0.23154 (12)	0.0244 (4)	
H13A	0.1799	0.9736	0.1903	0.029*	
H13B	0.2851	0.9971	0.1944	0.029*	
C14	0.40732 (16)	1.0840 (2)	0.38860 (16)	0.0436 (5)	
H14A	0.4320	1.1541	0.4422	0.065*	
H14B	0.4376	1.1086	0.3465	0.065*	
H14C	0.4221	0.9767	0.4101	0.065*	
O4A	0.2865 (2)	0.6950 (4)	0.0984 (2)	0.0223 (6)	0.882 (5)
O5A	0.1227 (3)	0.7245 (6)	0.0253 (2)	0.0350 (8)	0.882 (5)
C15A	0.19637 (16)	0.7028 (3)	0.09603 (17)	0.0182 (5)	0.882 (5)
C16A	0.29113 (18)	0.7026 (3)	0.00803 (15)	0.0350 (6)	0.882 (5)
H16A	0.3583	0.6962	0.0169	0.053*	0.882 (5)
H16B	0.2633	0.8007	−0.0225	0.053*	0.882 (5)
H16C	0.2549	0.6160	−0.0308	0.053*	0.882 (5)
O4B	0.1389 (17)	0.715 (4)	0.0255 (17)	0.018 (5)*	0.118 (5)
O5B	0.3045 (17)	0.707 (4)	0.114 (2)	0.028 (7)*	0.118 (5)
C15B	0.2218 (18)	0.697 (5)	0.104 (2)	0.045 (5)*	0.118 (5)
C16B	0.1512 (14)	0.732 (2)	−0.0598 (13)	0.045 (5)*	0.118 (5)
H16D	0.0883	0.7433	−0.1121	0.067*	0.118 (5)
H16E	0.1834	0.6394	−0.0695	0.067*	0.118 (5)
H16F	0.1905	0.8234	−0.0556	0.067*	0.118 (5)
C17	−0.00079 (17)	0.5767 (3)	0.2584 (2)	0.0491 (6)	
H17A	−0.0411	0.6694	0.2377	0.074*	
H17B	0.0215	0.5650	0.3258	0.074*	
H17C	−0.0383	0.4853	0.2270	0.074*	
C18	0.49909 (13)	0.4745 (2)	0.35923 (15)	0.0379 (5)	
H18A	0.5443	0.4075	0.3472	0.057*	
H18B	0.5008	0.4500	0.4204	0.057*	
H18C	0.5172	0.5832	0.3581	0.057*	

### Atomic displacement parameters (Å^2^)

**Table:**

	*U*^11^	*U*^22^	*U*^33^	*U*^12^	*U*^13^	*U*^23^
S1	0.0334 (2)	0.0158 (2)	0.0250 (2)	0.00124 (15)	0.01191 (19)	−0.00212 (15)
O1	0.0415 (7)	0.0212 (6)	0.0162 (6)	0.0016 (5)	0.0073 (5)	−0.0005 (4)
O2	0.0252 (7)	0.0440 (8)	0.0519 (9)	−0.0037 (6)	0.0221 (7)	−0.0092 (7)
O3	0.0268 (6)	0.0174 (6)	0.0292 (6)	0.0042 (4)	0.0187 (5)	0.0061 (5)
N1	0.0173 (6)	0.0205 (7)	0.0218 (7)	−0.0020 (5)	0.0025 (6)	0.0003 (5)
N2	0.0232 (7)	0.0200 (7)	0.0289 (8)	0.0031 (5)	0.0125 (6)	0.0038 (6)
C1	0.0255 (8)	0.0123 (7)	0.0182 (8)	−0.0006 (6)	0.0070 (7)	0.0008 (6)
C2	0.0227 (8)	0.0197 (8)	0.0286 (9)	0.0000 (6)	0.0122 (7)	0.0012 (6)
C3	0.0226 (8)	0.0161 (7)	0.0172 (7)	0.0009 (6)	0.0088 (6)	0.0021 (6)
C4	0.0341 (9)	0.0205 (8)	0.0222 (8)	0.0049 (7)	0.0153 (7)	0.0009 (6)
C5	0.0424 (10)	0.0229 (9)	0.0185 (8)	0.0027 (7)	0.0080 (8)	−0.0040 (6)
C6	0.0290 (9)	0.0268 (9)	0.0242 (9)	−0.0017 (7)	−0.0009 (8)	−0.0044 (7)
C7	0.0204 (8)	0.0228 (8)	0.0265 (9)	0.0011 (6)	0.0052 (7)	−0.0005 (7)
C8	0.0197 (7)	0.0137 (7)	0.0159 (7)	0.0007 (5)	0.0064 (6)	0.0017 (5)
C9	0.0202 (7)	0.0145 (7)	0.0174 (7)	−0.0001 (5)	0.0095 (6)	0.0002 (5)
C10	0.0273 (9)	0.0205 (8)	0.0329 (9)	0.0050 (6)	0.0162 (8)	0.0025 (7)
C11	0.0229 (8)	0.0135 (7)	0.0167 (7)	0.0015 (6)	0.0085 (6)	0.0022 (6)
C12	0.0297 (8)	0.0148 (7)	0.0153 (7)	−0.0006 (6)	0.0087 (7)	−0.0001 (6)
C13	0.0356 (9)	0.0159 (8)	0.0193 (8)	−0.0006 (6)	0.0091 (7)	0.0009 (6)
C14	0.0371 (11)	0.0313 (10)	0.0445 (12)	0.0036 (8)	−0.0007 (10)	−0.0062 (9)
O4A	0.0278 (14)	0.0245 (10)	0.0164 (12)	−0.0063 (10)	0.0109 (10)	−0.0028 (9)
O5A	0.0325 (15)	0.0456 (15)	0.0204 (10)	0.0042 (14)	0.0045 (10)	0.0055 (7)
C15A	0.0253 (12)	0.0130 (9)	0.0157 (10)	−0.0023 (10)	0.0080 (10)	0.0001 (7)
C16A	0.0484 (14)	0.0411 (13)	0.0268 (11)	−0.0108 (10)	0.0269 (10)	−0.0040 (9)
C17	0.0464 (12)	0.0363 (11)	0.0867 (18)	0.0122 (9)	0.0496 (13)	0.0174 (11)
C18	0.0222 (9)	0.0357 (11)	0.0391 (11)	−0.0067 (7)	−0.0038 (8)	−0.0004 (9)

### Geometric parameters (Å, °)

**Table:**

S1—C14	1.795 (2)	C11—C15B	1.57 (3)
S1—C13	1.8019 (17)	C12—C13	1.535 (2)
O1—C1	1.213 (2)	C12—H12A	0.9700
O2—C2	1.218 (2)	C12—H12B	0.9700
O3—C10	1.3706 (19)	C13—H13A	0.9700
O3—C9	1.4328 (18)	C13—H13B	0.9700
N1—C1	1.387 (2)	C14—H14A	0.9600
N1—C2	1.391 (2)	C14—H14B	0.9600
N1—C18	1.471 (2)	C14—H14C	0.9600
N2—C10	1.263 (2)	O4A—C15A	1.340 (3)
N2—C11	1.461 (2)	O4A—C16A	1.431 (4)
C1—C9	1.520 (2)	O5A—C15A	1.203 (4)
C2—C3	1.478 (2)	C16A—H16A	0.9600
C3—C8	1.395 (2)	C16A—H16B	0.9600
C3—C4	1.401 (2)	C16A—H16C	0.9600
C4—C5	1.380 (3)	O4B—C15B	1.336 (18)
C4—H4A	0.9300	O4B—C16B	1.416 (19)
C5—C6	1.386 (3)	O5B—C15B	1.189 (19)
C5—H5A	0.9300	C16B—H16D	0.9600
C6—C7	1.390 (3)	C16B—H16E	0.9600
C6—H6A	0.9300	C16B—H16F	0.9600
C7—C8	1.389 (2)	C17—H17A	0.9600
C7—H7A	0.9300	C17—H17B	0.9600
C8—C9	1.507 (2)	C17—H17C	0.9600
C9—C11	1.615 (2)	C18—H18A	0.9600
C10—C17	1.484 (3)	C18—H18B	0.9600
C11—C15A	1.520 (3)	C18—H18C	0.9600
C11—C12	1.542 (2)		
			
C14—S1—C13	100.99 (9)	C13—C12—H12A	109.4
C10—O3—C9	107.16 (12)	C11—C12—H12A	109.4
C1—N1—C2	124.12 (13)	C13—C12—H12B	109.4
C1—N1—C18	117.65 (15)	C11—C12—H12B	109.4
C2—N1—C18	118.05 (15)	H12A—C12—H12B	108.0
C10—N2—C11	107.72 (13)	C12—C13—S1	113.78 (11)
O1—C1—N1	121.45 (15)	C12—C13—H13A	108.8
O1—C1—C9	122.08 (15)	S1—C13—H13A	108.8
N1—C1—C9	116.08 (13)	C12—C13—H13B	108.8
O2—C2—N1	120.26 (16)	S1—C13—H13B	108.8
O2—C2—C3	122.63 (16)	H13A—C13—H13B	107.7
N1—C2—C3	116.99 (14)	S1—C14—H14A	109.5
C8—C3—C4	120.20 (15)	S1—C14—H14B	109.5
C8—C3—C2	121.07 (14)	H14A—C14—H14B	109.5
C4—C3—C2	118.72 (14)	S1—C14—H14C	109.5
C5—C4—C3	119.91 (16)	H14A—C14—H14C	109.5
C5—C4—H4A	120.0	H14B—C14—H14C	109.5
C3—C4—H4A	120.0	C15A—O4A—C16A	115.5 (3)
C4—C5—C6	119.80 (16)	O5A—C15A—O4A	124.6 (3)
C4—C5—H5A	120.1	O5A—C15A—C11	125.5 (2)
C6—C5—H5A	120.1	O4A—C15A—C11	109.9 (2)
C5—C6—C7	120.72 (16)	O4A—C16A—H16A	109.5
C5—C6—H6A	119.6	O4A—C16A—H16B	109.5
C7—C6—H6A	119.6	H16A—C16A—H16B	109.5
C8—C7—C6	119.95 (16)	O4A—C16A—H16C	109.5
C8—C7—H7A	120.0	H16A—C16A—H16C	109.5
C6—C7—H7A	120.0	H16B—C16A—H16C	109.5
C7—C8—C3	119.41 (15)	C15B—O4B—C16B	115 (2)
C7—C8—C9	121.87 (14)	O5B—C15B—O4B	129 (3)
C3—C8—C9	118.65 (14)	O5B—C15B—C11	118 (2)
O3—C9—C8	109.92 (12)	O4B—C15B—C11	112 (2)
O3—C9—C1	108.37 (12)	O4B—C16B—H16D	109.5
C8—C9—C1	112.76 (12)	O4B—C16B—H16E	109.5
O3—C9—C11	101.78 (11)	H16D—C16B—H16E	109.5
C8—C9—C11	113.19 (12)	O4B—C16B—H16F	109.5
C1—C9—C11	110.17 (12)	H16D—C16B—H16F	109.5
N2—C10—O3	118.36 (15)	H16E—C16B—H16F	109.5
N2—C10—C17	127.13 (16)	C10—C17—H17A	109.5
O3—C10—C17	114.50 (15)	C10—C17—H17B	109.5
N2—C11—C15A	109.81 (14)	H17A—C17—H17B	109.5
N2—C11—C12	108.00 (13)	C10—C17—H17C	109.5
C15A—C11—C12	110.16 (15)	H17A—C17—H17C	109.5
N2—C11—C15B	122.6 (9)	H17B—C17—H17C	109.5
C12—C11—C15B	102.6 (12)	N1—C18—H18A	109.5
N2—C11—C9	103.00 (12)	N1—C18—H18B	109.5
C15A—C11—C9	111.08 (15)	H18A—C18—H18B	109.5
C12—C11—C9	114.46 (12)	N1—C18—H18C	109.5
C15B—C11—C9	106.7 (15)	H18A—C18—H18C	109.5
C13—C12—C11	111.29 (13)	H18B—C18—H18C	109.5
			
C2—N1—C1—O1	165.16 (15)	C10—N2—C11—C15A	129.83 (18)
C18—N1—C1—O1	−9.9 (2)	C10—N2—C11—C12	−110.02 (15)
C2—N1—C1—C9	−21.8 (2)	C10—N2—C11—C15B	131.3 (18)
C18—N1—C1—C9	163.11 (14)	C10—N2—C11—C9	11.44 (17)
C1—N1—C2—O2	−179.30 (16)	O3—C9—C11—N2	−13.71 (14)
C18—N1—C2—O2	−4.2 (2)	C8—C9—C11—N2	104.20 (14)
C1—N1—C2—C3	−3.2 (2)	C1—C9—C11—N2	−128.52 (13)
C18—N1—C2—C3	171.80 (15)	O3—C9—C11—C15A	−131.21 (14)
O2—C2—C3—C8	−171.88 (16)	C8—C9—C11—C15A	−13.30 (19)
N1—C2—C3—C8	12.2 (2)	C1—C9—C11—C15A	113.98 (16)
O2—C2—C3—C4	9.5 (3)	O3—C9—C11—C12	103.26 (14)
N1—C2—C3—C4	−166.48 (14)	C8—C9—C11—C12	−138.83 (14)
C8—C3—C4—C5	−1.1 (2)	C1—C9—C11—C12	−11.55 (17)
C2—C3—C4—C5	177.51 (16)	O3—C9—C11—C15B	−144.0 (9)
C3—C4—C5—C6	0.3 (3)	C8—C9—C11—C15B	−26.1 (9)
C4—C5—C6—C7	0.8 (3)	C1—C9—C11—C15B	101.2 (9)
C5—C6—C7—C8	−1.1 (3)	N2—C11—C12—C13	−64.30 (16)
C6—C7—C8—C3	0.3 (2)	C15A—C11—C12—C13	55.63 (19)
C6—C7—C8—C9	177.18 (15)	C15B—C11—C12—C13	66.5 (13)
C4—C3—C8—C7	0.8 (2)	C9—C11—C12—C13	−178.36 (13)
C2—C3—C8—C7	−177.81 (15)	C11—C12—C13—S1	145.74 (12)
C4—C3—C8—C9	−176.15 (14)	C14—S1—C13—C12	61.63 (15)
C2—C3—C8—C9	5.2 (2)	C16A—O4A—C15A—O5A	−4.5 (5)
C10—O3—C9—C8	−108.91 (14)	C16A—O4A—C15A—C11	175.0 (2)
C10—O3—C9—C1	127.45 (13)	N2—C11—C15A—O5A	−8.1 (4)
C10—O3—C9—C11	11.32 (15)	C12—C11—C15A—O5A	−126.9 (4)
C7—C8—C9—O3	33.2 (2)	C15B—C11—C15A—O5A	178 (7)
C3—C8—C9—O3	−149.93 (13)	C9—C11—C15A—O5A	105.2 (4)
C7—C8—C9—C1	154.22 (15)	N2—C11—C15A—O4A	172.3 (2)
C3—C8—C9—C1	−28.89 (19)	C12—C11—C15A—O4A	53.5 (3)
C7—C8—C9—C11	−79.87 (18)	C15B—C11—C15A—O4A	−2(7)
C3—C8—C9—C11	97.02 (16)	C9—C11—C15A—O4A	−74.4 (3)
O1—C1—C9—O3	−28.3 (2)	C16B—O4B—C15B—O5B	7(7)
N1—C1—C9—O3	158.75 (13)	C16B—O4B—C15B—C11	−180 (2)
O1—C1—C9—C8	−150.20 (15)	N2—C11—C15B—O5B	163 (3)
N1—C1—C9—C8	36.83 (18)	C15A—C11—C15B—O5B	169 (11)
O1—C1—C9—C11	82.28 (18)	C12—C11—C15B—O5B	42 (4)
N1—C1—C9—C11	−90.69 (15)	C9—C11—C15B—O5B	−79 (4)
C11—N2—C10—O3	−4.9 (2)	N2—C11—C15B—O4B	−11 (4)
C11—N2—C10—C17	174.1 (2)	C15A—C11—C15B—O4B	−5(5)
C9—O3—C10—N2	−5.1 (2)	C12—C11—C15B—O4B	−132 (3)
C9—O3—C10—C17	175.80 (17)	C9—C11—C15B—O4B	107 (3)

### Hydrogen-bond geometry (Å, °)

**Table:**

*D*—H···*A*	*D*—H	H···*A*	*D*···*A*	*D*—H···*A*
C18—H18C···O2^i^	0.96	2.49	3.436 (2)	167.

Symmetry codes: (i) −*x*+1, *y*+1/2, −*z*+1/2.
